# Relationship between Anthropometric Factors, Gender, and Balance under Unstable Conditions in Young Adults

**DOI:** 10.1155/2013/850424

**Published:** 2013-01-16

**Authors:** Júlia Maria D'Andréa Greve, Mutlu Cuğ, Deniz Dülgeroğlu, Guilherme Carlos Brech, Angelica Castilho Alonso

**Affiliations:** ^1^Laboratory of Movement Studies (LEM), Institute of Orthopedics and Traumatology (IOT), Hospital das Clínicas (HC), School of Medicine, University of São Paulo, 05403-010 São Paulo, SP, Brazil; ^2^Physical Education and Sports Department, Cumhuriyet University, 58140 Sivas, Turkey; ^3^Diskapi Yildirim Beyazit Education and Research Hospital, Physical Medicine and Rehabilitation Clinic, Ankara, Turkey

## Abstract

The objective of this study was to evaluate the relationship between the anthropometric factors of height, body mass, body mass index and postural balance and to compare the balance indices between genders in the upright standing position, in healthy adult subjects under conditions of instability. Forty individuals were subjected to functional tests of body stability using the Biodex Balance System, and the resulting indices were correlated with body mass, height, and body mass index, and also compared between genders. Body mass was the main anthropometric factor that influenced variations in postural balance, with a high correlation between groups and with all variables. A linear regression analysis showed that body mass associated with BMI explained 66% of the overall stability, and body mass explained 59% of the anteroposterior stability index and 65% of the mediolateral stability index. In the female group, body mass explained 72% of the overall balance, 66% of the anteroposterior, and 76% of the medio-lateral stability index. Increased body mass requires greater movements to maintain postural balance. Height and BMI presented moderate correlations with balance. Women showed less movement than men on the Biodex Balance System.

## 1. Introduction

Balance is defined as the ability to maintain the body's center of mass within the base of support. The balance is maintained through the movement of body weight in different directions with safety, speed (response time), and coordination. Balance is dynamic and requires constant adjustments to adapt to external perturbations, through the use of vision, muscle activity, articular positioning and proprioception, and the vestibular system, all acting in concert [1, 2].

Balance evaluation tests that simulate functional activities are the most appropriate type of test to determine the contributions of the musculoskeletal, vestibular, and visual systems. Systems of maintaining postural balance can be affected by lesions, musculoskeletal, or neurological limitations, anthropometric factors, aging, use of medications, physical conditioning, and specific training (e.g., high impact sports), as well as extrinsic factors such as the type of shoes and the type of ground [3].

Many balance assessment methods exist, ranging from simple observation, clinical tests, scales, and posturographic measurements, to integrated assessment systems of greater complexity. All of them have advantages and limitations and can demonstrate different results with multiple interpretations, and this is exacerbated by the lack of consensus regarding which individual characteristics (especially anthropometric factors) should to be controlled for, so that quantitative evaluations can be considered reliable. This lack of consensus impedes the use of such tests in clinical practice as a safe tool for assessing the risk of falls and the results from therapeutic interventions [3–6].

Cote et al. [7] reported that balance measurements should be controlled in order to avoid errors in analyzing the results and highlighted anthropometric factors as important in this type of evaluation. Studies seeking normative data for controlling the variables that might influence balance assessments are needed in the scientific literature, since there is still no consensus regarding the influence of anthropometric measurements on postural balance. 

The objective of this study was to evaluate the relationship between the anthropometric factors of height, body mass, body mass index (BMI), and postural balance and to compare the balance indices between genders in the upright standing position, in healthy adult subjects under conditions of instability.

## 2. Method

This study was conducted in Diskapi Yildirim Beyazit Education and Research Hospital, Physical Medicine and Rehabilitation Clinic, Ankara, Turkey in partnership with the Laboratory of Movement Studies, University of São Paulo Medical School, Brazil. The study was approved by the Middle East Technical University (METU) School of Natural and Applied Science Ethics Committee (no. 00.00/126/78-1597).

This was a descriptive observational, cross-sectional study, without intervention. Forty volunteers (twenty-five males and fifteen females) were evaluated. The mean age was 21.7 ± 1.4 years (20–27), with a mean body mass of 65.7 ± 12.4 kg (43–94), mean height of 170 cm (157–185), and mean BMI of 22.5 ± 3.5 kg/m^2^(16.1–31.4). For the males, the mean age was 21.5 ± 1.4 years (20–27), with a mean body mass of 71.2 ± 9.1 kg (57–90), mean height of 173.8 ± 0.1 cm (161–185) and mean BMI of 23.5 ± 2.8 kg/m^2^ (18.8–29.3). For the females, the mean age was 21.8 ± 1.1 years (20–24), with a mean body mass of 56.5 ± 12.1 kg (43–94), mean height of 164 ± 0.5 cm (157–173), and mean BMI of 20.8 ± 3.9 kg/m^2^(16.1–31.4).

The inclusion criteria were as follows: (a) signing of a free and informed consent statement; (b) age between 20 and 30 years; (c) no physical activity for a minimum of six months (according to the physical activity readiness questionnaire); (d) absence of neurological, cardiovascular, metabolic, rheumatic or vestibular diseases; (e) no injuries or previous surgery on the legs; and (f) absence of knee or ankle clinical instability. Individuals who required more than three attempts to accomplish the test were excluded from the study, but none of the individuals met this exclusion criterion.

All the volunteers answered a questionnaire requesting personal information, in order to identify the inclusion criteria. Thirty-seven volunteers (92.5%) had a dominant right leg (kicking side), and three (7.5%) had a dominant left leg. 

The same evaluator performed all the anthropometric measurements (body mass, height, and BMI) and the balance test. The balance test was performed using the Biodex Balance System (BBS) (Neurocom International, Inc. Clackamas, OR, USA) with the level 2 stability protocol, which allows an inclination of up to 20° in the horizontal plane in all directions. Stability varies according to the resistance level equipment (from level 8—more stable, to level 1—less stable) [1, 7]. Three stability indices were calculated as follows: antero-posterior stability index—represented the variance of foot platform displacement in degrees, from level, for motion in the sagittal plane. Mediolateral stability index- represented the variance of foot platform displacement in degrees, from level, for motion in the frontal plane and overall stability index (sum of the first two)—represented the variance of foot platform displacement in degrees in all motions during a test. It was the angular excursion of the patient's center of gravity. A high number was indicative of a lot of movement during a test with static measures; it was the angular excursion of the patient's center of gravity. The arithmetic means of the results were calculated from the three tests. The subjects were tested with their eyes open at all times.

### 2.1. Positioning

The subjects were asked to step onto the BSS platform and take a comfortable bipedal standing position, while maintaining slight flexion of the knees (15°), looking straight ahead, and folding the arms across the chest. The subjects were tested barefoot at all times. 

The platform was released, and the patients were instructed to balance themselves, keeping the indicator at the center of the target on the screen. When the patient was capable of keeping the indicator in the center of the target on the screen (balanced position) without hand support, the foot position was recorded using the platform rail.

### 2.2. Test

Once the subjects had been positioned, they were instructed not to move their feet until the end of each measurement. Changes were recorded in relation to the center of the platform. Two 20-second measurements were made separated by one-minute intervals. The result was the arithmetic mean of the two measurements, which was supplied automatically by the equipment. 

### 2.3. Statistical Analysis

The Kolmogorov-Smirnov test was used to analyze the gender distribution and the continuous variable in the parametric and nonparametric tests. As the data presented normal distribution, the paired Student's *t*-test was used. The SPSS 20.0 software for Windows was used, adopting a level of significance of *P* ≤ 0.05.

Pearson's coefficient was used to evaluate the correlation between anteroposterior, medio-lateral and general stability (sum of the first two) and the anthropometric measurements (body mass, height, and BMI) of each subject. The correlation was high when *R* ≥ 0.7, moderate when 0.5 ≤ *R* < 0.7, low when 0.1 ≤ *R* ≤ 0.5, and null when *R* = 0.

To analyze the linear regression model, all the variables that presented *P* ≤ 0.20 in the correlation coefficient analysis were selected. These were then placed in order from lowest to highest *P* value. The multiple modeling processes followed the stepwise forward selection method, in which the variables were added one by one, according to their ranking. The variables that resulted in *P* ≤ 0.05 remained in the model.

## 3. Results

The females presented better stability indices (general, antero-posterior and medio-lateral) than the males (Table 1). 

The correlation analysis between the anthropometric variables and postural balance in the whole group and divided according to gender is described in Table 2. 

Regression analysis on the anthropometric variables in relation to postural balance in the whole group and divided according to gender is described in Table 3.

## 4. Discussion

Identifying factors that influence balance can help improve the accuracy of diagnosis and quality of treatment and rehabilitation (indication of specific exercises) and is fundamental for preventing falls and incapacities [5, 8]. The BBS is a reliable and reproducible method for evaluating antero-posterior and medio-lateral movements from the body's center of mass that can be made while maintaining postural stability [9].

Anthropometric variables influence the stability limits of the organism and can affect the motor strategies relating to balance control [5]. Some anthropometric variables, such as body mass, are directly related to postural balance; however, the majority of works evaluate specific groups, such as obese adolescents [4], the elderly [1, 10, 11], and athletes [12]. Yet few studies have reported on individuals with normal body mass or overweight who were subjected to conditions of instability. 

 The male group demonstrated stronger correlations for overall, antero-posterior, and medio-lateral stability index with BMI. Women had moderate correlations for all variables with BMI. In the whole group evaluation, the correlations between BMI and the stability indexes were also moderate in regression analysis associated with body mass, which explained 66% of overall balance. Greve et al. [6] showed that in young adult males, the higher the BMI, worst postural balance, needing more postural adjustments to maintain balance in single leg stance. Thus, their data were similar to the current study. The women of this study had lower BMI and body mass than the men, but had similar height, which may have contributed to reducing the strength of correlation and regression with BMI. Other studies conducted on individuals with normal or slightly higher than normal BMI have shown low correlations between body mass and balance [13, 14]. The differences between these studies are likely related to the evaluation techniques. It seems that in situations of instability, body mass presented greater impairment of balance.

The male group had stronger correlations for overall, antero-posterior and medio-lateral stability with body mass. Women had moderate correlations for all variables with body mass. In the whole group evaluation, the correlations between body mass and the stability indexes were also stronger. The difference between genders can slightly alter the behavior of the inverted pendulum model. The inverted pendulum model should be carefully applied to study postural control. In order to avoid any misunderstanding during the analysis of the inverted pendulum model applied in the quiet standing posture, it is necessary to consider the mass distribution in the calculus for the position of the center of mass [5, 13, 15].

The majority of studies indicate that there was a direct relationship between obesity and increased postural instability, as evaluated by means of various tools and methods [4, 8, 14–17]. In the present study, body mass presented a high correlation with the stability indices; that is, there was a need for greater movements to maintain postural balance. This finding was similar to that of Ledin and Odkvist [8] who demonstrated that a 20% increase in body mass reduced the ability to make adjustments in response to external perturbations in the orthostatic position, with a consequent increase in postural instability. Hue et al. [18] found that body mass was responsible for more than 50% of balance at speed and Chiari et al. [13] demonstrated a strong correlation between body mass, antero-posterior movements, and the area of detachment. In both of these studies, a force platform was used in the evaluations. Other authors have reported that greater postural adjustments are necessary to maintain an erect posture when there is a buildup of adipose tissue, thus causing a reduction in balance and an increase in injuries and falls [4, 8, 17].

Due to the high degree of correlation between balance and body mass, we can safely infer that the mechanical factor of body mass inertia requires greater musculoskeletal force to balance it against the force of gravity, and therefore, to maintain balance. Obese individuals require greater movement from the center of gravity to remain in the orthostatic position. This study showed that only a high body mass can contribute towards decreasing the balance and occurrences of falls in situations of instability.

The male and female groups, and the group overall, had moderate correlations for all variables with height. In the regression analysis for the male group, height explained over half of the overall and medio-lateral stability index, associated with body mass. Ku et al. [19] found that BMI has an impact on postural control in both the bipedal and unipodal stance. These findings corroborate the data in the literature. There is a consensus that the greater the height is, the worse the balance. Berger et al. [20] and Alonso et al. [21] stated that ankle displacements and the response of the gastrocnemius increased with increasing height. Allard et al. [22] and Lee and Lin [23] reported that ectomorph (lanky) individuals present greater postural sway than do endomorph or mesomorph individuals, and they attributed this to the higher position of the center of mass. Other studies have found that body stability is inversely related to the height of the center of gravity and that, for this reason, posturography measurements are affected by individuals' anthropometric characteristics [5, 18].

Chiari et al. [13] and Kejonen et al. [5] suggested that height and body mass could be affected by the total load of movements that occur at the top of the “inverted pendulum,” in which when standing upright on two feet, the pivot is the ankle joint and the support base is the interface between the body and the ground (geometry). These authors recommended that for this reason, these variables should always be evaluated as a set. 

Since the correlations found were similar, it was not possible to determine which movements were the greatest instability factors: medio-lateral (movements of the pelvis and lower limbs) or antero-posterior (movements of the trunk).

Comparing the genders, we saw that women showed less movement on the BBS than men did, and these findings were similar to those of Rozzi et al. [24] who evaluated basketball and American football players using the same equipment. In another study involving children, it was observed that girls presented better postural balance than boys [23].

This could be due to anthropometric factors (greater in men), but other factors such as neuromuscular (flexibility) and neurophysiologic (processing of inferences), as well as the habit of using higher heels, may also account for the differences. 

The BBS is a simple system that enables rapid evaluation and, when used with certain criteria, it may be of assistance for quantifying alterations in balance. It can also be used for dysfunction training. 

These results safely suggest that incorporating the evaluation of body composition in patients with equal BMI can help understand and use this correlation in order to prevent falls and other incapacities of obese patients.

## 5. Conclusion

Increased body mass required greater movement to maintain postural balance. Height and BMI presented moderate correlations with balance. Women showed less movement than men on the BBS.

## Figures and Tables

**Table 1:** Comparison between general, anteroposterior, and mediolateral stability indexes (mean and SD) distributed according gender.

Stability index	Male (*N* = 25) Mean (SD)	Female (*N* = 15) Mean (SD)	*P *
Overall balance	6.6 (±2.8)	3.7 (±2.7)	0.003*
Anteroposterior	4.9 (±2.0)	2.9 (±2.0)	0.004*
Mediolateral	4.5 (±2.0)	2.5 (±1.9)	0.004*

*P ≤ 0.05—Student's *t*-test.

**Table 2:** Correlation (*r* value) between the general, anteroposterior, and mediolateral stability indexes and height (cm), body mass (kg), and BMI (kg/m^2^).

Stability index	Height R (*P* value)	Body mass R (*P* value)	BMI R (*P* value)
General (*N* = 40)			

Overall balance	0.624 (0.000)*	0.808 (0.000)*	0.647 (0.000)*
Anteroposterior	0.598 (0.000)*	0.779 (0.000)*	0.627 (0.000)*
Mediolateral	0.631 (0.000)*	0.813 (0.000)*	0.650 (0.000)*

Male (*N* = 25)			

Overall balance	0.423 (0.117)	0.864 (0.000)*	0.804 (0.000)*
Anteroposterior	0.408 (0.132)	0.829 (0.000)*	0.767 (0.000)*
Mediolateral	0.449 (0.094)	0.885 (0.000)*	0.822 (0.000)*

Female (*N* = 15)			

Overall balance	0.530 (0.000)*	0.680 (0.000)*	0.411 (0.041)*
Anteroposterior	0.488 (0.013)*	0.636 (0.001)*	0.391 (0.053)*
Mediolateral	0.534 (0.006)*	0.688 (0.000)*	0.415 (0.039)*

*P ≤ 0.05—Pearson's coefficient.

General group: all the anthropometric variables presented moderate to strong positive correlations with the postural balance variables.

Male group: the variables of body mass and BMI presented strong positive correlations with the postural balance variables.

Female group: all the anthropometric variables presented weak to moderate positive correlations with the postural balance variables.

**Table 3:** Linear regression analysis between postural balance and anthropometric variables.

Stability index	Height *β* (*P* value)	Body mass *β* (*P* value)	BMI *β* (*P* value)	Adjusted *r* ^2^
General (*N* = 40)				

Overall balance		0.292 (<0.001)	−0.365 (0.058)	0.668
Anteroposterior	—	0.140 (<0.001)	—	0.596
Mediolateral	—	0.143 (<0.001)	—	0.652

Male (*N* = 25)				

Overall balance	15.003 (0.046)	0.175 (0.001)	—	0.512
Anteroposterior	—	0.143 (0.001)	—	0.378
Mediolateral	10.900 (0.042)	0.128 (0.001)	—	0.526

Female (*N* = 15)				

Overall balance	—	0.192 (<0.001)	—	0.727
Anteroposterior	—	0.136 (<0.001)	—	0.663
Mediolateral	—	0.136 (<0.001)	—	0.767

*r* ^2^ = linear regression coefficient.

General group: body mass associated with BMI explained 66% of the stability index for overall balance, and body mass explained 59% of the anteroposterior stability index and 65% of the mediolateral stability index.

Male group: body mass associated with height explained 51% of the overall balance, body mass explained 37% of the anteroposterior stability index, and body mass associated with height explained 52% of the mediolateral stability index.

Female group: body mass explained 72% of the overall balance, 66% of the anteroposterior stability index, and 76% of the mediolateral stability index.

## References

[1] Prado JM, Stoffregen TA, Duarte M (2007). Postural sway during dual tasks in young and elderly adults. *Gerontology*.

[2] Alonso AC, Greve JMDA, Camanho GL (2009). Evaluating the center of gravity of dislocations in soccer players with and without reconstruction of the anterior cruciate ligament using a balance platform. *Clinics*.

[3] Alonso AC, Brech GC, Bourquin AM, Greve JMDA (2011). The influence of lower-limb dominance on postural balance. *Sao Paulo Medical Journal*.

[4] McGraw B, McClenaghan BA, Williams HG, Dickerson J, Ward DS (2000). Gait and postural stability in obese and nonobese prepubertal boys. *Archives of Physical Medicine and Rehabilitation*.

[5] kejonen P, Kauranen K, Vanharanta H (2003). The relationship between anthropometric factors and body-balancing movements in postural balance. *Archives of Physical Medicine and Rehabilitation*.

[6] Greve JMDA, Alonso AC, Bordini ACPG, Camanho GL (2007). Correlation between body mass index and postural balance. *Clinics*.

[7] Cote KP, Brunet ME, Gansneder BM, Shultz SJ (2005). Effects of pronated and supinated foot postures on static and dynamic postural stability. *Journal of Athletic Training*.

[8] Ledin T, Odkvist LM (1993). Effects of increased inertial load in dynamic and randomized perturbed posturography. *Acta Oto-Laryngologica*.

[9] Cachupe WJC, Shifflett B, Kahanov L, Wughalter EH (2001). Reliability of biodex balance system measures. *Measurement in Physical Education and Exercise Science*.

[10] Mainenti MRM, Rodrigues EC, Oliveira JF, Ferreira AS, Dias CM, Silva ALS (2011). Adiposity and postural balance control: correlations between bioelectrical impedance and stabilometric signals in elderly Brazilian women. *Clinics*.

[11] Swanenburg J, de Bruin ED, Favero K, Uebelhart D, Mulder T (2008). The reliability of postural balance measures in single and dual tasking in elderly fallers and non-fallers. *BMC Musculoskeletal Disorders*.

[12] Alonso AC, Bronzatto-Filho E, Brech GC, Moscoli FV (2008). Estudo comparativo do equilíbrio postural entre atletas de judô e indivíduos sedentários. *Revista Brasileira de Biomecânica*.

[13] Chiari L, Rocchi L, Cappello A (2002). Stabilometric parameters are affected by anthropometry and foot placement. *Clinical Biomechanics*.

[14] Molikova R, Bezdickova M, Langova K (2006). The relationship between morphological indicators of human body and posture. *Biomedical Papers of the Medical Faculty of the University Palacký, Olomouc, Czechoslovakia*.

[15] Loram ID, Gollee H, Lakie M, Gawthrop PJ (2011). Human control of an inverted pendulum: is continuous control necessary? Is intermittent control effective? Is intermittent control physiological?. *Journal of Physiology*.

[16] Goulding A, Jones IE, Taylor RW, Piggot JM, Taylor D (2003). Dynamic and static tests of balance and postural sway in boys: effects of previous wrist bone fractures and high adiposity. *Gait and Posture*.

[17] Berrigan F, Simoneau M, Tremblay A, Hue O, Teasdale N (2006). Influence of obesity on accurate and rapid arm movement performed from a standing posture. *International Journal of Obesity*.

[18] Hue O, Simoneau M, Marcotte J (2007). Body weight is a strong predictor of postural stability. *Gait and Posture*.

[19] Ku PX, Abu Osman NA, Yusof A, Abas WAW (2012). Biomechanical evaluation of the relationship between postural control and body mass index. *Journal of Biomechanics*.

[20] Berger W, Trippel M, Discher M, Dietz V (1992). Influence of subjects’ height on the stabilization of posture. *Acta Oto-Laryngologica*.

[21] Alonso AC, Luna NMS, Mochizuki L, Barbieri F, Santos S, Greve JMDA (2012). The influence of anthropometric factors on postural balance: the relationship between body composition and posturographic measurements in young adults. *Clinics*.

[22] Allard P, Nault ML, Hinse S, LeBlanc R, Labelle H (2001). Relationship between morphologic somatotypes and standing posture equilibrium. *Annals of Human Biology*.

[23] Lee AJY, Lin WH (2007). The influence of gender and somatotype on single-leg upright standing postural stability in children. *Journal of Applied Biomechanics*.

[24] Rozzi SL, Lephart SM, Gear WS, Fu FH (1999). Knee joint laxity and neuromuscular characteristics of male and female soccer and basketball players. *American Journal of Sports Medicine*.

